# Understanding the Food Waste Reduction Intentions of Consumers in Turkiye Through the Value–Attitude–Behavior Framework

**DOI:** 10.3390/foods15122127

**Published:** 2026-06-12

**Authors:** Şaziye Ecem Örkü, Merve Nur Uçak, Elif Şahin, Ece Öneş, Meryem Kahrıman, Cansu Gençalp, Murat Baş, Perim Fatma Türker

**Affiliations:** 1 Department of Nutrition and Dietetics, Faculty of Health Sciences, Acibadem Mehmet Ali Aydinlar University, İstanbul 34755, Türkiye; merve.ucak@acibadem.edu.tr (M.N.U.); ece.ones@acibadem.edu.tr (E.Ö.); meryem.kahriman@acibadem.edu.tr (M.K.); cansu.gencalp@acibadem.edu.tr (C.G.); murat.bas@acibadem.edu.tr (M.B.); perim.turker@acibadem.edu.tr (P.F.T.); 2 Department of Nutrition and Dietetics, Faculty of Health Sciences, Gedik University, İstanbul 34876, Türkiye; elif.sahin@gedik.edu.tr; 3 Department of Nutrition and Dietetics, Graduate School of Health Sciences, Acibadem Mehmet Ali Aydinlar University, İstanbul 34755, Türkiye

**Keywords:** value–attitude–behavior model, theory of planned behavior, food waste reduction, anticipated guilt, social norms, attitude to reduce food waste

## Abstract

Food loss and waste are a major global problem as reflected in the United Nations Sustainable Development Goal 12. Households constitute the primary source of food waste worldwide. The development of effective solutions depends on a comprehensive understanding of consumer attitudes and behaviors. This cross-sectional study used the Value–Attitude–Behavior (VAB) hierarchy to examine consumers’ food waste reduction intentions. It was conducted on individuals in Turkiye via an online survey. The results showed that consumers’ hedonic value and attitudes were positively associated with food waste reduction intentions. The strongest associations with intentions were observed for anticipated guilt, attitude toward reducing food waste, and hedonic value. Furthermore, education level and household size showed significant effects on food waste reduction intentions. In conclusion, these findings based on the VAB model showed the central role of anticipated guilt in shaping food waste reduction intentions, suggesting that emotionally driven intervention strategies may be more effective than approaches focusing solely on attitudes.

## 1. Introduction

The food loss and waste (FLW) is defined as a decrease in the quantity or quality of edible food intended for human consumption by the Food and Agriculture Organization (FAO) of the United Nations. In other words, it represents the removal of food intended for human consumption from the supply chain [1]. It is also crucial to distinguish between food loss and food waste. Food loss results from the decisions and actions of suppliers at earlier stages of the supply chain, such as harvesting, storage, transportation, etc. In contrast, food waste typically occurs at the retail and consumer levels [2].

FLW significantly impacts economic, environmental, and social costs [3]. According to the FAO, one-third of the food produced for human consumption is either lost or wasted. It corresponds to 1.3 billion tons and about $1 trillion in economic losses annually, while generating about 8% of global greenhouse gas emissions [4]. Food waste (FW) in the European Union is estimated at 88 million tonnes annually, one-fifth of the total food produced, with economic costs of €143 billion. Two-thirds of such a loss was attributed to households, indicating their impact on FLW [5]. In the United States, 62 million tonnes of food are lost or wasted, corresponding to $218 trillion a year [6]. A study estimated FLW in Turkiye to be 26 million tonnes per year [7]. A countrywide FLW report indicates that food losses are predominant during agricultural production, with fruits and vegetables representing the most frequently lost commodities [8].

At the environmental level, FLW also impacts climate, water foodprint, land use, and biodiversity. In 2007, the global blue water footprint of agriculture-related food wastage was about 250 km^3^, roughly 3.6 times that of the U.S. In addition, nearly 1.4 billion hectares of land were utilized to produce food that was never consumed, an area exceeding Canada and India combined [9]. Such inefficiency drives deforestation, diminishes biodiversity, and accelerates ecosystem degradation, each of which carries significant long-term environmental consequences [10]. According to the Waste and Resources Action Program (WRAP), until 2024, FLW was responsible for 8–10% of total greenhouse gas (GHG) emissions. If FW were considered a country, it would rank as the world’s third-largest emitter of GHGs, following China and the United States [11].

FLW also threatens food security; even as it causes global demand for food to rise continuously, millions of people remain undernourished. Recently, the FAO has estimated that approximately 828 million people face food insecurity, with one-third of global food production being lost or wasted annually [12]. Similarly, in the United States, almost 40% of all food produced goes uneaten, while more than 34 million people struggle with food insecurity [13]. This widespread wastage results in missed opportunities to feed vulnerable populations. If FLW could be reduced or repurposed, it could help reduce global hunger and improve food security. For instance, recovering just one-quarter of the food currently wasted would be enough to feed approximately 870 million people, solving global hunger [14].

Food loss is primarily observed in the supply chains of developing countries, whereas FW is more commonly experienced in highly urbanized and economically developed regions. Approximately 56% of global FLW occurs in developed countries [1,15]. FLW varies in magnitude through the stages of a food supply chain. Based on World Resource Institute analysis, 35 percent of global FLW occurs at consumption level [16]. According to the “2021 Food Waste Index Report” published by the United Nations Environment Program (UNEP) in 2019, 931 million tons of food were wasted at this level, and proportionately 61% came from households, 26% from food service, and 13% from retail, corresponding to 17% of total food production [17]. A 2024 FWIR report indicates that the problem continues to rise; in 2022, the total global food waste reached 1.05 billion tonnes, approximately 132 kg/person and representing nearly one-fifth of all food available for consumption. Similarly, a majority of FW occurred at the household level (60%), followed by food services (28%) and retail (12%) [18]. In addition, the 2024 FWIR data show that food waste is not only a problem in developed countries, as household food waste levels vary by only 7 kg per person across high-, upper-, and lower-middle-income countries. This highlights the critical importance of consumer behavior in addressing food waste reduction.

A growing recognition of FLW as a major global problem is reflected in its inclusion in the United Nations Sustainable Development Goal (SDG) 12, wherein a dedicated target focuses on minimizing such losses. Target 12.3 aims to halve global per capita FW and reduce food losses throughout the production and supply chains by 2030 [19]. Effective solutions to reduce FLW require an understanding of how the stages of the food supply chain are connected.

Despite the growing body of research on food waste, the psychological mechanisms underlying consumers’ food waste reduction intentions remain insufficiently understood. Previous studies have predominantly relied on cognitive frameworks, while the role of emotionally driven factors has received comparatively less attention. In particular, limited research has examined how hedonic value, sense of community, social norms, and anticipated guilt interact within the Value–Attitude–Behavior framework to shape food waste reduction intentions. Addressing this gap, the present study proposes an integrated model to better explain consumers’ intentions to reduce food waste.

## 2. Theoretical Framework

A diverse set of strategies has been implemented to mitigate FLW. They must account for all stages of the food supply chain, spanning from agricultural production to household consumption [16]. Policy recommendations for reducing FW should be based on an analysis of its root causes. Considering the extent of food wasted by households, understanding the drivers and implementing consumer-targeted campaigns and educational programs represents a crucial strategy [20]. While knowledge and awareness are vital for decreasing household FW, Richetin et al. (2012) note that they may not be decisive in changing behavior [21]. Although the literature recognizes reduction behavior as a critical component in addressing FW, research on consumer behavior related to FW remains limited [22]. Previous studies suggest that FW is driven by a range of causes and behaviors, reflecting its complex nature. Some studies have employed the Theory of Planned Behavior (TPB) to analyze FW behavior [23]. According to the TPB, the intention to act is considered the primary determinant of behavior [24]. However, recent reports have found its explanatory power limited, as, from a cognitive perspective, it fails to consider emotion-related factors essential for understanding FW behavior [25,26,27]. A recently published meta-analysis compiled 56 studies that examined FW behavior through TPB and demonstrated that its theoretical limitations in explaining FW reduction behavior [28]. Nevertheless, this limitation does not imply that TPB-related constructs are irrelevant in explaining food waste behavior. Rather, integrating socio-cognitive determinants such as attitudes and social norms with value-based and emotion-related factors may provide a more comprehensive understanding of food waste reduction intentions.

Demographic factors are also vital determinants in driving FW behavior. However, the literature presents conflicting findings on this issue. In terms of age, studies show that older adults (50 and above) tend to waste less food than younger individuals [29]. In contrast, young consumers exhibit greater awareness and concerns regarding FW [30]. Education is another determining factor, with studies indicating that higher education levels are associated with less FW. However, the effectiveness of education depends on infrastructural conditions [31]. Gender has also been identified as an important factor influencing food-related attitudes and waste behaviors at the consumer level [32]. On the other hand, although women are generally more involved in meal planning, factors such as marital status and household composition can influence portioning and meal preparation practices, which may in turn contribute to food waste depending on family size and household dynamics [33].

Food waste behavior is also influenced by broader contextual factors beyond individual-level psychological determinants. These include access to food retail environments, characteristics of short supply chains, and changes in food distribution and consumption systems [34]. However, the present study focuses specifically on individual-level psychological determinants within the Value–Attitude–Behavior framework.

### 2.1. Value–Attitude–Behavior (VAB) Model

Researchers argue that individuals are guided by certain values, which are reflected in their behavior and goals. Based on the idea that values play a central role in the formation of personal identity, they are considered internal influencers of behavior and attitudes [35]. The VAB model is a cognitive hierarchy that examines the relationship between values, attitudes, and behavior, developed by Homer and Kahle [36]. It assumes that values directly affect attitudes, which eventually influence behaviors. In this situation, it is assumed that values may indirectly impact behaviors. It has been widely used for understanding environmental behaviors over the last few decades. For example, studies on sustainability/pro-environmental behavior [36,37], FW [25,38], and organic food consumption [39] included the VAB model as a method.

### 2.2. Hypothesis Development

Values vary in relative importance; when a person attributes significance to a particular value, behavior is shaped in the associated direction. In terms of pro-environmental behaviors, recent studies identified four values: altruistic, hedonistic, egoistic, and biospheric [40]. Among these value orientations, hedonic value was prioritized in the present study because the previous food waste literature has more frequently associated food-related decision making and waste reduction behavior with pleasure-oriented and experiential motivations [25,38]. Consumers with HVs prioritize pleasure as their defining goal. Pro-environmental food preference may also be associated with pleasure. Therefore, it may be positively related to attitudes and behaviors [41,42]. Based on the literature mentioned, we hypothesize as follows:

**H1a.**  *HV is positively associated with anticipated guilt (ANG).*

**H1b.**  *HV is positively associated with social norms (SN).*

**H1c.**  *HV is positively associated with attitude to reduce FW (ATT).*

Sense of community (SOC) is a term coined by McMillan and Davis [43] and has four elements: membership, influence, integration and fulfillment of needs, and shared emotional connection [43]. SOCs play a regulatory role in social environments by strengthening social ties and identity [44]. Belonging to a particular group/community also evokes collective action. In terms of FW reduction behavior, SOC is found to be a positive motive through attitudes. Individuals with a stronger sense of community tend to internalize collective values and shared responsibilities more strongly. As a result, behaviors perceived as socially or environmentally irresponsible, such as food waste, may evoke stronger anticipated guilt due to concerns about violating collective expectations and social responsibility norms [27,45]. Based on the literature discussed above, our hypotheses were as follows:

**H2a.**  *SOC is positively associated with ANG.*

**H2b.**  *SOC is positively associated with SN.*

**H2c.**  *SOC is positively associated with ATT.*

It is widely accepted that attitudes have a critical role in shaping human behavior [46]. A positive relationship also occurs between pro-environmental attitudes and behaviors [47]. However, even when individuals are positive toward a behavior, they do not always engage in it. To address this attitude–behavior gap, the literature has examined a range of individual, social, and contextual factors as moderators of pro-environmental behavior, including social norms, environmental concern, trust, price, etc. [47,48,49]. From a cognitive perspective, social norms are an effective tool to promote behavior by motivating individuals toward collective pro-environmental goals [50]. In addition to cognitive factors, emotions are also thought to be an influential tool promoting such behavior. Evidence indicates that anticipated guilt can accurately predict consumer behaviors related to FW [27]. Feeling guilty markedly affects consumer intention to reduce FW. It usually stems from cultural standards that prioritize FW reduction and optimal resource utilization [51]. ATT refers to individuals’ evaluations of FW and recycling behavior; positive attitudes encourage FW reduction behavior [52].

**H3.**  *ANG is positively associated with an intention to reduce food waste.*

**H4.**  *SN is positively associated with FW reduction intention.*

**H5.**  *ATT is positively associated with FW reduction intention.*

**Figure 1:**
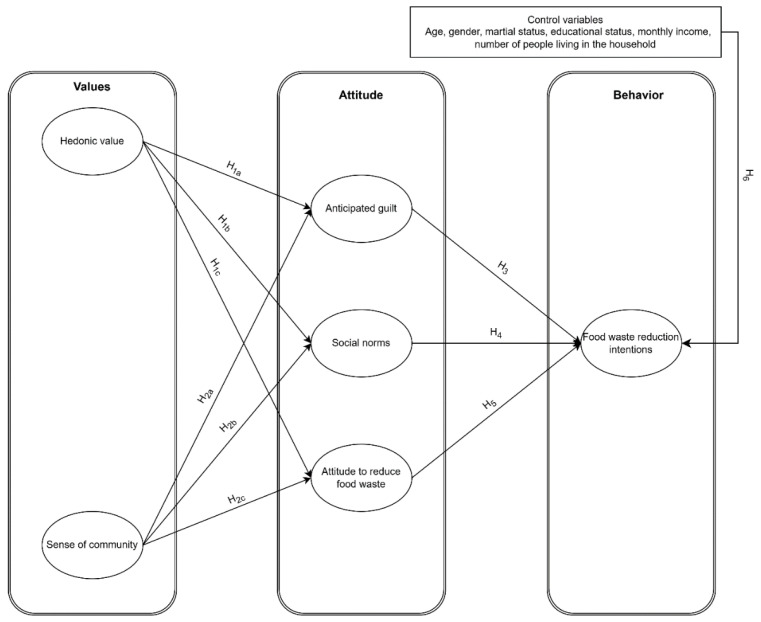
The proposed research model.

Therefore, this study aimed to better understand the relationship between HV, SOC, social norms, anticipated guilt, attitude to reduce FW, and FW reduction intentions based on the VAB model. The proposed research model is shown in Figure 1.

## 3. Research Methods

### 3.1. Data Collection and Study Sampling

Participants were selected using snowball sampling, a non-probabilistic sampling method. During the data collection process, a survey link created via Google Forms was shared on social media platforms, and initial participants were asked to share the survey with their networks. This method allowed for a gradual expansion of the sample, and voluntary participation was prioritized. Individuals aged 18 and over were included. Before starting, the participants were requested to complete an informed consent form, and approval (2024-8/325) was obtained from the Acibadem Mehmet Ali Aydinlar University Medical Research Ethics Committee.

When administering a scale, the sample size must be sufficiently large to represent the complete population. Muthén and Muthén (2002) recommended a sample size of at least 5–10 times the number of items in the scale [53]. While higher ratios are generally preferred for greater statistical robustness, a minimum ratio of 5:1 is considered acceptable, particularly in exploratory studies.

In line with this recommendation, the minimum sample size required for this study was calculated based on the lower limit of this range. Given that the scale consists of 18 items, the goal was to include a minimum of 90 to 180 participants in the study.

The study included 1392 participants: 44.1% male and 55.9% female. The mean age of the men was 34.27 ± 10.20 years, 70.5% were single, 50.2% worked in the private sector, 41% held a postgraduate degree, 55.9% reported purchasing some environmentally friendly (green) products during their monthly shopping, 46.7% paid some attention to whether the products they bought were sustainable or environmentally friendly, 53.6% paid close attention to whether the products they purchased were healthy, 44.6% sometimes had problems with food leftovers, and 29.5% sometimes shared leftovers (Table 1).

The mean age of the women was 30.03 ± 8.53 years, 74.2% were single, 48.6% worked in the private sector, 39.7% had postgraduate education, 57.6% reported purchasing some environmentally friendly (green) products during their monthly shopping, 48.8% paid some attention to whether the products they bought were sustainable or environmentally friendly, 54.2% paid great attention to whether the products they purchased were healthy, 43.8% sometimes had problems with food leftovers, and 28.7% sometimes shared leftovers.

### 3.2. Measurement Scale

The questionnaire used in the study consisted of two parts. The first part included questions about the demographic information of the participants, and the second part included questions investigating specific variables related to the intention to reduce food waste. The second part consisted of items measuring 6 variables related to the intention to reduce food waste: hedonic value, sense of community, anticipated guilt, social norms, attitude to reduce food waste, and food waste reduction intentions (Table 2). These items were adapted from previous studies [27,54,55,56] by making the necessary changes to fit the context of food waste reduction, as reported by Habib et al. (2023) [25]. In total, 18 items were measured using a five-point Likert scale ranging from 1 (strongly disagree) to 5 (strongly agree).

### 3.3. Data Analysis

Descriptive statistics for categorical variables are presented as frequencies and percentages. The Shapiro–Wilk Test confirmed whether numerical variables followed a normal distribution. Descriptive statistics for numerical variables are presented as mean ± standard deviation ($\bar{X}$ ± SD) for normally distributed data and as median (min–max) for non-normally distributed data.

Convergent validity accounts for the construct validity of the scale [57]. It means that the statements regarding variables are related to each other and the factors they constitute [58].

For convergent validity, all composite reliability (CR) values for a scale must be greater than the average verified and extracted values, the AVE must be >0.5, the standardized factor loadings of the items must be >0.5, and the CR must be >0.7 [57]. The AVE is evaluated by dividing the sum of the squares of the item loadings for a factor by the number of items [59]. The coefficient Cronbach’s Alpha was calculated to assess the reliability of the scales studied.

For discriminant validity, two new values must be calculated. The maximum squared variance (MSV) is the square of the highest variance shared by a factor with any other factor. The average shared square variance (ASV) is obtained by dividing the sum of the squares of the variance shared by a factor with other factors by the number of shared variances. To speak of discriminant validity, the condition MSV < AVE; ASV < MSV must be met, and the square root of AVE must be greater than the correlation between the factors [58].

Structural equation modeling (SEM) was employed to test the hypothesized relationships and mediation effects. The model was estimated using the maximum likelihood (ML) estimator. Model fit was assessed using multiple goodness-of-fit indices, including the Comparative Fit Index (CFI), Tucker–Lewis Index (TLI), Root Mean Square Error of Approximation (RMSEA), and Standardized Root Mean Square Residual (SRMR). Demographic variables were incorporated as control variables in the analysis to assess their effects on food waste reduction intention. SEM analyses were conducted using the lavaan and sem packages of R Project v3.6.1 (R Core Team, Vienna, Austria) [60].

Pearson’s correlation coefficient was used to examine the relationships between scales for normally distributed data. The criteria used to interpret the correlation coefficient included- “<0.2: very weak correlation,” “0.2–0.4: weak correlation,” “0.4–0.6: moderate correlation,” “0.6–0.8: high correlation,” and “>0.8: very high correlation” [61].

Multiple regression analysis was used to test the effects of variables. It is the mathematical equivalence of the effects of two or more dependent variables [62]. In all calculations and interpretations, the statistical significance level was considered *p* < 0.05, *p* < 0.01, *p* < 0.001, and hypotheses were established in two directions. Statistical analyses of the data were performed using SPSS v26 (IBM Inc., Chicago, IL, USA) and the R Project v3.6.1 (R Core Team, Vienna, Austria) package programs.

## 4. Results

This correlation analysis (Table 3) indicated that FW reduction intentions were positively associated with all factors (*p* < 0.001). The strongest relations with intentions were observed for anticipated guilt (r = 0.594), attitude toward reducing FW (r = 0.546), and hedonic value (r = 0.514).

When convergent validity was examined (Table 4), the standardized factor loadings ranged between 0.821 and 0.980, the CR values exceeded the recommended threshold of 0.7, and all AVE values were above 0.5. These findings indicated that the scale demonstrated convergent validity. Regarding reliability, CR and Cronbach’s-α values exceeded the recommended threshold of 0.7, indicating robust internal consistency across all constructs.

Examination of discriminant validity (Table 5) showed that the AVE values for all constructs exceeded their corresponding MSV and ASV values.

Table 6 summarizes the descriptive statistics of the study variables, indicating that FW reduction intentions exhibited the maximum mean score (16.87 ± 5.14), whereas attitude to reduce FW displayed the lowest (8.40 ± 2.05).

**Figure 2:**
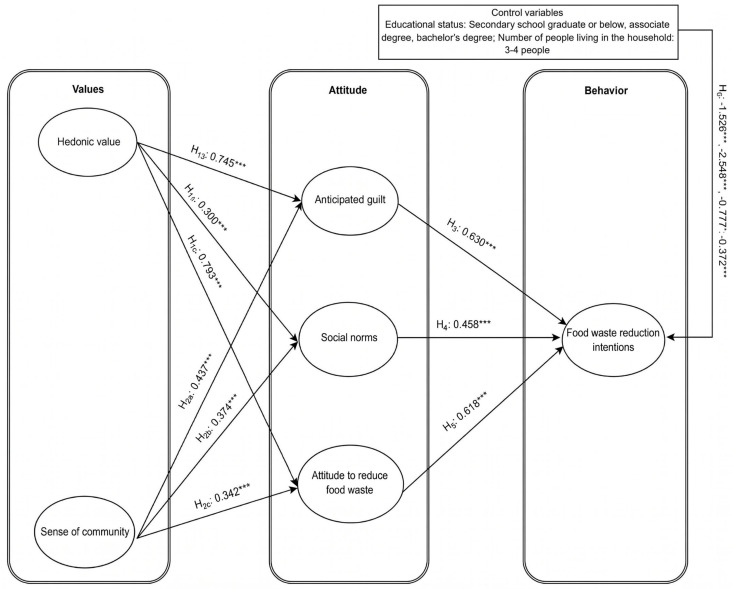
Results of hypothesis testing (* *p* < 0.05; *** *p* < 0.001).

Hedonic value and SOC directly and significantly affected anticipated guilt, social norms, and attitudes toward reducing FW (*p* < 0.001 for all). Specifically, hedonic value had positive effects of 74.5% on anticipated guilt, 30% on social norms, and 79.3% on attitudes. SOC exhibited positive impacts of 43.7% on anticipated guilt, 37.4% on social norms, and 34.2% on attitudes toward reducing FW (Table 7; Figure 2).

Anticipated guilt, social norms, and attitudes toward reducing FW each showed direct and significant effects on FW reduction intentions (*p* < 0.001). Anticipated guilt had a positive influence of 63%, social norms of 45.8%, and attitudes of 61.8% on intentions (Table 8; Figure 2).

When examining the effects of demographic variables on FW reduction intentions, age, gender, marital status, and monthly income were found to have no significant influence on intention scores (*p* > 0.05). In contrast, education level and household size showed statistically significant effects on FW reduction intentions (*p* < 0.05; *p* < 0.001).

According to the regression coefficients, individuals educated up to lower secondary or associate degree had intention scores that were 1.526 and 2.548 points lower than those of postgraduate graduates. Bachelor’s degree holders, however, scored 0.777 points higher than postgraduate graduates. In addition, individuals living in households with 3–4 members had intention scores 1.146 points greater than those living in households with 1–2 persons. No significant variation was observed for households with ≥five members (*p* = 0.459) (Table 9; Figure 2).

## 5. Discussion

This study provides an integrated psychosocial perspective on consumers’ food waste reduction intentions by combining emotional, social, and attitudinal determinants. The research combines the explanatory power of the VAB model with core components of the TPB, proposing a comprehensive and complementary approach to understanding food waste-associated behaviors. The VAB model, built upon the Value–Attitude–Behavior hierarchy, offers a robust theoretical foundation for explaining individuals’ behavioral tendencies [63] and its utilization in FW studies has increased in recent years [25]. The findings particularly highlight the role of hedonic value and anticipated guilt in shaping food waste reduction intentions.

This study positions HV as a multifaceted psychological construct central to behavioral processes aimed at reducing FW. HV may indirectly shape sustainable behavioral tendencies through emotional (anticipated guilt) and cognitive/normative (ATT and SN) mechanisms. In this context, HV is not a direct determinant of intention; instead, it may reinforce intention by strengthening sensitivity to guilt, social expectations, and positive attitudes toward food waste reduction. These findings are consistent with previous VAB-based studies suggesting that hedonic value may strengthen food waste reduction intentions through emotional and attitudinal pathways. 

These findings suggest that hedonic value may function as an important psychological motivator in strengthening food waste reduction intentions. Supporting the hedonic experience is an essential factor while designing interventions to reduce FW. Future research should examine whether the VAB model’s predictive power varies across food categories. For example, hedonic value may more strongly predict intention for fresh produce (associated with quality, health, pleasure) compared to shelf-stable items (associated with practicality, budgeting). Tailored interventions accounting for category-specific consumer psychology could enhance effectiveness.

The remarkable influence of moral and social factors on the intention to reduce FW represents a key finding. Among the variables included in the model, anticipated guilt emerged as one of the strongest factors associated with food waste reduction intentions. This result suggests that individuals’ internalized emotional responses to wasteful behavior can markedly motivate sustainable actions. Guilt-related emotions may also encourage individuals to align their behaviors with perceived social and environmental responsibilities.

Social norms also appeared to play an important role in shaping food waste reduction intentions. This observation indicates that individuals are significantly affected by societal expectations and social approval mechanisms, with the reduction in FW viewed as a behavior partially considered “socially expected.” The influence of SN may also be strengthened by connections to hedonic value and a SOC, suggesting that the potential for social approval or disapproval serves a regulatory function in individual behavior.

The SOC has emerged as a higher-level construct that specifically supports emotional (anticipated guilt) and normative (social norms) processes. These findings suggest that a stronger sense of community may enhance individuals’ emotional and normative sensitivity toward food waste reduction. Although SOC was not directly associated with attitudes, it serves as a crucial background mechanism that enhances intention via guilt and SN.

These findings are consistent with previous research highlighting the role of moral–emotional processes in sustainable behavior. Attiq et al. (2021) indicate that negative emotions, including guilt and awareness of consequences, significantly predict reduction, reuse, and recycling behaviors in households and young consumers [64,65]. Similarly, moral obligation and anticipated pride, a positive self-conscious emotion, markedly impact intention, demonstrating that moral emotions function through self-reward and guilt avoidance [66]; pride and guilt are powerful indicators of intention [67]. Additionally, longitudinal data indicate that regret produced more variable outcomes in a context-dependent manner, whereas the effect of guilt was very stable over time [51]. Taken together, these studies imply that individual dispositions and features of the consumption context influence emotional processes.

Overall, the findings suggest that the intention to reduce FW is significantly influenced by moral emotions (guilt, pride, and regret), a SOC, and SN. In addition to being an environmental issue, people consider waste a moral and social concern, as evidenced by the intersection of emotional reactions and social expectations. As a result, people are more likely to develop a stronger intention to reduce waste in social environments where normative pressure and a sense of belonging to a community are prominent.

The findings further support the idea that food waste reduction intention is shaped by both emotional and normative-cognitive processes. In particular, anticipated guilt appears to play an important role in motivating sustainable behavioral intentions.

Social norms also appeared to contribute to food waste reduction intentions, suggesting that waste reduction may be perceived as a socially encouraged behavior. Attitudes toward reducing food waste similarly supported behavioral intention, highlighting the continued relevance of key TPB components in explaining food waste-related behaviors.

These results mostly align with earlier research outlining the factors influencing food waste reduction intentions. According to [68], the extended TPB model demonstrates that attitude, subjective norm, moral norm, anticipated regret, and self-identity all significantly explain intention. Similarly, waste reduction practices are influenced by SN and evaluative attitudes in norm-sensitive and habit-based consumer segments [56]. Furthermore, a thorough systematic review has verified that the most trustworthy direct predictors of intention are attitude, SN, and self-conscious feelings like regret or guilt [33].

Complementary research also highlights the important role of moral–emotional components, demonstrating that within extended norm-activation frameworks, intention was significantly influenced by personal beliefs and self-efficacy. Evidence from Chinese households also shows that moral attitudes and anticipated guilt are among the most vital factors associated with intentions to reduce food waste, underscoring the transformative role of emotional processes when moral responsibility is activated [69].

One notable finding of this study is that anticipated guilt appeared to be a stronger predictor than several traditional TPB-related components. Such a result supports the Theory of Interpersonal Behavior’s emphasis on emotional and personal norm-based components; it implies that adding emotional variables to TPB-based models can greatly enhance their explanatory power when it relates to FW. Emotional factors, especially guilt-based mechanisms, consistently exert greater effects on intention than cognitive predictors, according to systematic evidence [33].

When all this evidence is evaluated together, it is evident that the intention to reduce FW is fueled not only by cognitive processes but also by intense moral–emotional evaluations. This observation suggests that emotion- and identity-based interventions may be more effective than those that merely provide information or improve general awareness.

Taken together, these global trends align with evidence collected at the national level. Similar dynamics have been documented in Turkiye. According to reports, moral norms acted as a mediating factor, and subjective norms and perceived behavioral control were strong predictors in TPB-based models, whereas attitude was comparatively weak [70]. Similarly, in an extended TPB model, subjective norms and perceived control were the most effective variables regarding intention and behavior [71]. These findings imply that social norm-based effects predominate in the formation of intentions in Turkiye, particularly due to the collective nature of family structures, the social environment’s directive role, and intense community pressure. This pattern may reflect the relatively stronger influence of social and community-based factors in the Turkish context.

To better understand the factors influencing the intention to reduce FW, this study included demographic characteristics as control variables. The results demonstrate that intention is not significantly impacted by age, gender, marital status, or income level. Nevertheless, important connections emerged between these factors and psychosocial constructs that can influence behavior. For instance, women and younger individuals appeared to be more sensitive to social expectations related to food waste reduction. This trend suggests that different demographic subgroups may experience varying degrees of the impact of SN on behavioral intention.

Household size and educational status are the two demographic factors that markedly influence intention. The idea that higher education is linked to greater environmental awareness and sustainability consciousness is supported by postgraduate and graduate degree holders having a stronger intention to reduce waste [72]. In a similar vein, higher intention levels among individuals living in households with 3–4 members may reflect a greater sense of shared responsibility and conformity to social norms. These results are in line with earlier studies showing that household size influences waste behaviors [73].

These findings show that demographic factors can influence behavioral intentions by indirectly affecting psychosocial mechanisms, even though they may not always function as direct predictors. Wastage-related behaviors are influenced by age, gender, education, and household size through intermediary variables such as perceived behavioral control and environmental concern [26].

A systematic review of more than 100 empirical studies offered a more thorough evaluation of how demographic factors affect FW behavior. While sociodemographic factors like age, gender, income, educational status, and household size frequently do not directly determine FW, they do have a remarkable impact on behavioral outcomes through indirect pathways [74]. According to the review, these elements influence waste through practical and psychological mechanisms, including planning abilities, stock management, shopping habits, degree of environmental concern, and sensitivity to SN. Additionally, demographic profiles need to be considered in both research and intervention designs because younger people, larger households, and groups with lower educational levels tend to have higher waste inclinations.

Taken together, these findings suggest that rather than directly influencing FW behavior, demographic factors function as important contextual elements shaping psychosocial processes. To improve behavioral impact, policymakers and practitioners believe that customizing interventions to demographic segments like education level, age, and household structure is essential. Household-based approaches and communication strategies tailored to educational levels can greatly boost the efficacy of FW reduction programs, particularly in societies like Turkiye with strong family structures.

### 5.1. Theoretical Implications

These findings indicate that enriching the VAB model with emotional and normative components enhances explanatory power in the field of household FW compared to using the classical TPB alone. Specifically, the dominant effect of anticipated guilt on intention suggests the necessity of extended models centered on personal norms and self-conscious emotions in FW research. Furthermore, in the context of Turkiye, the decisive role of SN and household structure underscores the importance of systematically integrating the cultural context into behavioral intention models.

### 5.2. Practical Implications

In terms of application, the findings show that interventions aimed at reducing FW should focus not only on cognitive information transfer but also on emotional (guilt, pride) and hedonic components. Simultaneously emphasizing the moral dimension of waste and the “feel-good” effect (satisfaction from sustainable behavior) in campaigns can more robustly impact intention. Findings related to household size and educational status suggest that programs in Turkiye—especially those targeting multi-member and highly educated households and emphasizing intra-family collective decision-making processes and community norms—may be more effective.

At the same time, food waste reduction strategies should avoid placing the entire moral responsibility on consumers through excessive guilt- or shame-based messaging. Although anticipated guilt and emotional engagement emerged as important predictors of intention in the present study, consumers’ ability to reduce food waste is also influenced by structural and contextual factors beyond individual control. Economic limitations, time constraints, irregular work schedules, limited storage capacity, and modern convenience-oriented lifestyles may contribute to food waste despite positive environmental attitudes and intentions. Therefore, interventions should combine consumer-focused educational and emotional approaches with broader improvements in food supply, retail, storage, and distribution systems. Policies supporting practical food planning resources and more efficient supply-chain practices may help reduce the intention–behavior gap reported in previous food waste research.

## 6. Conclusions

This study advances the understanding of FW reduction behavior by offering an integrated psychosocial perspective grounded in the Value–Attitude–Behavior framework and supported by key constructs of the TPB. The findings demonstrate that FW reduction intentions are shaped through a hierarchical structure in which underlying values influence attitudes and, ultimately, behavioral intentions. In particular, anticipated guilt, social norms, and attitudes emerged as the most salient direct predictors of intention, while hedonic value and SOC function as important underlying factors operating through emotional and normative pathways.

The findings also suggest that FW is not merely a matter of rational decision making but rather a behavior deeply embedded in emotional responses and social expectations. These findings support the view that incorporating moral–emotional constructs into behavioral models may enhance explanatory power beyond traditional cognitive approaches. Furthermore, the findings suggest that demographic characteristics influence intention indirectly by shaping these psychosocial mechanisms rather than acting as primary determinants.

Overall, this study contributes to the environmental behavior literature by demonstrating the importance of integrating emotional, social, and value-based dimensions in explaining sustainable consumption practices. From a practical perspective, the results underline that interventions aimed at reducing FW should move beyond awareness-based strategies and instead leverage emotional engagement, social norms, and community-based approaches to foster meaningful behavioral change.

### 6.1. Applicability of the Model

The present findings suggest that the VAB framework may provide a useful approach for understanding household food waste reduction intentions in contexts where emotional, social, and community-related factors influence consumer behavior. In particular, the model may be applicable in societies characterized by strong family structures, collective social norms, and increasing environmental awareness, such as Turkiye. However, the applicability of the model may vary across different cultural, economic, and policy contexts. Future cross-cultural studies are needed to evaluate whether the observed psychosocial pathways remain consistent across populations with different social structures and sustainability practices.

### 6.2. Limitations and Future Directions

This study has several limitations. First, its cross-sectional design limits causal interpretation of the observed associations. Longitudinal and experimental studies are needed to better evaluate causal pathways between psychosocial factors and food waste reduction behavior. Second, the study relied on self-reported intentions rather than objectively measured food waste behavior. Future studies should therefore include behavioral assessments such as household food waste audits or purchasing records. Third, the online survey method may have introduced sampling bias toward younger and more digitally engaged individuals. More diverse sampling strategies may improve representativeness in future research. Finally, the findings are based on a Turkish sample and may not be fully generalizable to populations with different cultural or socioeconomic characteristics. Cross-cultural validation studies are therefore recommended.

## Figures and Tables

**Table 1:** Demographic characteristics of the participants.

	Men		Women		Total	
	*n*	%	*n*	%	*n*	%
Age (year) ($\bar{X}\pm SD$)	34.27 ± 10.20		30.03 ± 8.53		31.90 ± 9.54	
Marital Status						
Single	433	70.5	577	74.2	1010	72.6
Married	181	29.5	201	25.8	382	27.4
Occupation						
Civil servant	73	11.9	84	10.8	157	11.3
Private sector employees	308	50.2	378	48.6	686	49.3
Freelance	38	6.2	48	6.2	86	6.2
Employer	10	1.6	16	2.1	26	1.9
Housewife	14	2.3	21	2.7	35	2.5
Retired	29	4.7	27	3.5	56	4.0
Student	130	21.2	188	24.2	318	22.8
Unemployed/unemployed	12	2.0	16	2.1	28	2.0
Education Level						
Primary school/middle school graduate	13	2.1	16	2.1	29	2.1
Secondary school (high school) graduates	70	11.4	140	18.0	210	15.1
Associate degree graduate	38	6.2	44	5.7	82	5.9
Bachelor’s degree graduate	241	39.3	269	34.6	510	36.6
Postgraduate (master’s, doctorate, specialization)	252	41.0	309	39.7	561	40.3
Environmentally Friendly (Green) Product Purchase Rate in Monthly Shopping						
None/Very little	43	7.0	44	5.7	87	6.3
Little	105	17.1	120	15.4	225	16.2
Some	343	55.9	448	57.6	791	56.8
A lot	105	17.1	141	18.1	246	17.7
Very much	18	2.9	25	3.2	43	3.1
Attention to Sustainability and Environmentally Friendly Products Purchased						
None/Very little	65	10.6	69	8.9	134	9.6
Little	107	17.4	136	17.5	243	17.5
Some	287	46.7	380	48.8	667	47.9
A lot	134	21.8	169	21.7	303	21.8
Very much	21	3.4	24	3.1	45	3.2
Attention to the Health of the Products Purchased						
None/Very little	24	3.9	25	3.2	49	3.5
Little	26	4.2	29	3.7	55	4.0
Some	145	23.6	177	22.8	322	23.1
A lot	329	53.6	422	54.2	751	54.0
Very much	90	14.7	125	16.1	215	15.4
Monthly Income Status						
My income is less than my expenses.	227	37.0	301	38.7	528	37.9
My income is equal to my expenses.	195	31.8	238	30.6	433	31.1
My income is greater than my expenses.	192	31.3	239	30.7	431	31.0
Number of People Living in the House						
1−2 people	229	37.3	287	36.9	516	37.1
3–4 people	323	52.6	413	53.1	736	52.8
5 people and above	62	10.1	78	10.0	140	10.1
Self-Definition Status of the Person						
I live alone.	74	12.1	88	11.3	162	11.6
I am single and live with my child(ren).	5	0.8	7	0.9	12	0.9
I am unmarried and live with family members.	297	48.4	399	51.3	696	50.0
I live with roommates.	38	6.2	58	7.5	96	6.9
I live with my spouse/partner, and we have children.	91	14.8	99	12.7	190	13.6
I live with my spouse/partner, and we do not have children.	109	17.8	127	16.3	236	17.0
Having Food Leftovers Problem						
Yes	154	25.1	200	25.7	354	25.4
No	186	30.3	237	30.5	423	30.4
Sometimes	274	44.6	341	43.8	615	44.2
Increased Frequency of Leftover Sharing						
Never	163	26.5	177	22.8	340	24.4
Occasionally	130	21.2	171	22.0	301	21.6
Sometimes	181	29.5	223	28.7	404	29.0
Frequently	96	15.6	141	18.1	237	17.0
Always	44	7.2	66	8.5	110	7.9

**Table 2:** Variables and related items evaluated in the study.

Variable	Item
Hedonic Value [52]	I feel happy when I reduce household food waste. I feel pleasant when I reduce household food waste.
Sense of Community [27]	I feel a sense of community with the people living in my suburbs/neighborhood. I feel a sense of community with the people living in my city. I feel a sense of community with the people living in my country.
Anticipated Guilt [27]	I feel guilty when I waste household food as it has an adverse effect on the environment. I feel guilty when I waste household food as it has severe negative implications on the economy and society. I feel ashamed when I waste household food as it has a negative impact on our environment.
Social Norm [53]	Most people who are important to me think I should reduce the household food waste. Most people who are important to me would want me to reduce the household food waste. People whose opinions I value would prefer me to reduce the household food waste.
Attitude to Reduce Food Waste [54]	In my opinion, wasting household food is an extremely negative thing. In my opinion, wasting household food is an extremely unpleasant thing.
Food Waste Reduction Intentions [27]	In the near future, I intend to encourage colleagues and friends to reduce household food waste. I plan to take part in activities related to recycling household food waste. I will take part in household food waste recycling activities advocated in social media in the near future. I plan to promote recycling of household food waste to my friends, family, and peers. I will encourage my friends, colleagues and family to reduce the household food waste.

**Table 3:** Correlation coefficients among the study variables.

		HV	SOC	ANG	SN	ATT
SOC	s	0.300				
	*p*	<0.001 ***				
ANG	s	0.624	0.389			
	*p*	<0.001 ***	<0.001 ***			
SN	s	0.255	0.383	0.342		
	*p*	<0.001 ***	<0.001 ***	<0.001 ***		
ATT	s	0.692	0.296	0.693	0.241	
	*p*	<0.001 ***	<0.001 ***	<0.001 ***	<0.001 ***	
FWRI	s	0.514	0.441	0.594	0.468	0.546
	*p*	<0.001 ***	<0.001 ***	<0.001 ***	<0.001 ***	<0.001 ***

s = Spearman correlation; *** *p* < 0.001. ANG: anticipated guilt; ATT: attitude to reduce food waste; FWRI: food waste reduction intentions; HV: hedonic value; SN: social norms; and SOC: sense of community.

**Table 4:** Convergent validity and reliability.

Constructs	Items	SFL	CR	AVE	Cronbach’s α
HV	HV1	0.977	0.977	0.955	0.952
	HV2	0.977			
SOC	SOC1	0.885	0.940	0.828	0.895
	SOC2	0.959			
	SOC3	0.883			
ANG	ANG1	0.947	0.959	0.886	0.934
	ANG2	0.950			
	ANG3	0.926			
SN	SN1	0.873	0.939	0.837	0.892
	SN2	0.947			
	SN3	0.902			
ATT	ATT1	0.980	0.979	0.960	0.959
	ATT2	0.980			
FWRI	FWRI1	0.821	0.948	0.784	0.931
	FWRI2	0.914			
	FWRI3	0.877			
	FWRI4	0.912			
	FWRI5	0.901			

ANG: anticipated guilt; ATT: attitude to reduce food waste; AVE: average variance extracted; CR: composite reliability; FWRI: food waste reduction intentions; HV: hedonic value; SFL: standardized factor loadings; SN: social norms; and SOC: sense of community.

**Table 5:** Discriminant validity.

	CR	AVE	MSV	ASV	HV	SOC	ANG	SN	ATT	FWRI
HV	0.977	0.955	0.629	0.356	1.000					
SOC	0.940	0.828	0.206	0.161	0.387	1.000				
ANG	0.959	0.886	0.581	0.373	0.745	0.437	1.000			
SN	0.939	0.837	0.210	0.130	0.300	0.374	0.358	1.000		
ATT	0.979	0.960	0.382	0.359	0.793	0.342	0.762	0.291	1.000	
FWRI	0.948	0.784	0.397	0.307	0.584	0.454	0.630	0.458	0.618	1.000

ANG: anticipated guilt; ATT: attitude to reduce food waste; ASV: average shared variance; AVE: average variance extracted; CR: composite reliability; FWRI: food waste reduction intentions; HV: hedonic value; MSV: maximum shared variance; SFL: standardized factor loadings; SN: social norms; and SOC: sense of community.

**Table 6:** Descriptive statistics of the variables.

Construct	Items	$\bar{X}\pm SD$	Median (Min–Max)
HV	2	8.45 ± 1.89	9 (2–10)
SOC	3	8.92 ± 3.48	9 (3–15)
ANG	3	11.81 ± 3.01	12 (3–15)
SN	3	9.42 ± 3.56	10 (3–15)
ATT	2	8.40 ± 2.05	9 (2–10)
FWRI	5	16.87 ± 5.14	17 (5–25)

**Table 7:** The effects of hedonic value and sense of community on anticipated guilt, social norms, and attitudes toward reducing food waste.

	β	*t*-Value	*p*-Value
HV → ANG	0.745	41.591	<0.001 ***
HV → SN	0.300	11.704	<0.001 ***
HV → ATT	0.793	48.582	<0.001 ***
SOC → ANG	0.437	18.090	<0.001 ***
SOC → SN	0.374	15.026	<0.001 ***
SOC → ATT	0.342	13.578	<0.001 ***

*** *p* < 0.001. ANG: anticipated guilt; ATT: attitude to reduce food waste; HV: hedonic value; SN: social norms; and SOC: sense of community.

**Table 8:** The effects of anticipated guilt, social norms, and attitudes on food waste reduction intentions.

	β	*t*-Value	*p*-Value
ANG → FWRI	0.630	30.261	<0.001 ***
SN → FWRI	0.458	19.185	<0.001 ***
ATT → FWRI	0.618	29.321	<0.001 ***

*** *p* < 0.001. ANG: anticipated guilt; ATT: attitude to reduce food waste; SN: social norms; and FWRI: food waste reduction intentions.

**Table 9:** Effects of participants’ demographic characteristics on food waste reduction intention.

	Unstandardized Coefficients				95.0% Confidence Interval for B	
	B	SE	*t*	*p*	Lower-Bound	Upper-Bound
Constant	16.525	0.628	26.327	<0.001 ***	15.294	17.756
Age (year)	−0.014	0.015	−0.898	0.369	−0.044	0.016
(Ref: Woman)						
Man	−0.240	0.278	−0.863	0.389	−0.786	0.306
Marital status (Ref: Married)						
Single	0.493	0.326	1.514	0.130	−0.146	1.132
Education level (Ref: Postgraduate)						
Secondary school or below	−1.526	0.416	−3.673	<0.001 ***	−2.341	−0.711
Associate degree	−2.548	0.610	−4.179	<0.001 ***	−3.745	−1.352
Bachelor’s degree	0.777	0.311	2.501	0.012 *	0.168	1.386
Income (Ref: My income is higher than my expenses)						
My income is less than my expenses.	0.125	0.370	0.338	0.735	−0.601	0.851
My income equals my expenses.	0.143	0.354	0.403	0.687	−0.551	0.836
Number of people living in the house (Ref: a family of 1–2 people)						
3–4	1.146	0.296	3.868	<0.001 ***	0.565	1.726
>5	−0.372	0.502	−0.740	0.459	−1.357	0.614

SE: standard error. * *p* < 0.05; and *** *p* < 0.001.

## Data Availability

The original contributions presented in this study are included in the article. Further inquiries can be directed to the corresponding author.

## Abbreviations

The following abbreviations are used in this manuscript:

VAB	Value–Attitude–Behavior
FLW	Food Loss And Waste
FAO	Food and Agriculture Organization
FW	Food Waste
WRAP	Waste And Resources Action Program
GHG	Greenhouse Gas
UNEP	United Nations Environment Program
SDG	Sustainable Development Goal
TPB	Theory Of Planned Behavior
HV	Hedonic Value
ANG	Anticipated Guilt
SN	Social Norms
SOC	Sense Of Community
ATT	Attitude
CR	Composite Reliability
MSV	Maximum Squared Variance
ASV	Average Shared Square Variance
SEM	Structural Equation Modeling
AVE	Average Variance Extracted
FWRI	Food Waste Reduction Intentions
SFL	Standardized Factor Loadings
